# Pomalidomide improves the effectiveness of CAR-T treatment in the relapsed and refractory multiple myeloma or B-cell leukemia/lymphoma with extramedullary disease

**DOI:** 10.1097/BS9.0000000000000184

**Published:** 2024-03-01

**Authors:** Jie Zhao, Hui Yang, Junnan Ge, Linyu Li, Qiong Yao, Shaolong He, Qiujuan Zhu, Ruiui Ren, Chunrui Li, Liangming Ma, Weiwei Tian, Jia Wei

**Affiliations:** a Shanxi Bethune Hospital, Shanxi Academy of Medical Sciences, Tongji Shanxi Hospital, Third Hospital of Shanxi Medical University, Taiyuan, 030032, China; b Tongji Hospital, Tongji Medical College, Huazhong University of Science and Technology, Wuhan, 430030, China; c Hebei Taihe Chunyu Biotechnology Co. Ltd., Shijiazhuang, Hebei 050000, China; d Sino-German Joint Oncological Research Laboratory, Shanxi Bethune Hospital, Shanxi Academy of Medical Sciences, Taiyuan, Shanxi 030032, China; e Immunotherapy Research Center for Hematologic Diseases of Hubei Province, Wuhan, Hubei 430000, China

**Keywords:** B-cell malignancy, CAR-T, Extramedullary disease, Multiple myeloma, Pomalidomide

## Abstract

Relapsed and refractory multiple myeloma (RRMM) and B-cell leukemia/lymphoma with extramedullary disease (EMD) have poor prognosis and high mortality, lack of effective therapeutic approaches. We reported for the first time that 6 patients with malignant hematological diseases with EMD received chimeric antigen receptor (CAR)-T treatment combined with pomalidomide, and CAR-T cells were treated with pomalidomide in vitro to determine its killing activity and cytokine secretion. Three patients with RRMM were given B cell maturation antigen (BCMA)-CAR-T therapy. All 3 patients with B-cell leukemia/lymphoma received CD19/22-CAR-T sequential infusion. There were no treatment-related deaths. The maximum overall response rate (ORR) was 100%. Median follow-up was 211.5 days (75–407 days). Three patients (50%) experienced cytokine release syndrome, all of which were grade 1, and no neurotoxicity was observed. In vitro experiments showed that the killing activity did not differ significantly between BCMA-CAR-T cells with and without pomalidomide (10, 25, or 50 μg/mL) in 8226/U266 cell cocultures (*P* > .05). Tumor necrosis factor (TNF)-α and interferon (IFN)-γ secretion was significantly higher from 8226 and Raji cells cocultured with BCMA-CAR-T and cluster of differentiation (CD)19-CAR-T cells (*P* < .05). Based on the cocultures, adding pomalidomide significantly promoted IFN-γ and TNF-α secretion (*P* < .05). Based on the above clinical and in vitro studies demonstrating the co-administration of pomalidomide with CAR-T cell treatment demonstrated favorable tolerability and therapeutic effectiveness in RRMM or B-cell leukemia/lymphoma.

## 1. INTRODUCTION

Relapsed and refractory multiple myeloma (RRMM) and B-cell leukemia/lymphoma with extramedullary disease (EMD) remain challenging and indicate a poor prognosis. At present, chimeric antigen receptor (CAR)-T cell treatment has received approval for the management of RRMM and B-cell malignancies that have relapsed. This therapy specifically targets the cluster of differentiation (CD) 19 molecule (CD19) or the B-cell maturation antigen (BCMA).^1–3^ Nevertheless, individuals diagnosed with EMD frequently encounter early recurrence and observe restricted curative outcomes when subjected to CAR-T cell treatment.^4,5^ There are several potential contributors to this phenomenon, including T cell depletion during in vivo transport, functional impairment of CAR-T cells, and suppression of the tumor microenvironment (TME). Among them, the TME critically impacts T-cell activity, persistence, and migration.^6^ Hence, enhancing the TME and preserving T cell functionality emerges as a prospective approach towards improving the CAR-T cell treatments in the management of EMD.

It has been shown that pomalidomide, a third-generation immunomodulatory medication, is beneficial in controlling extramedullary multiple myeloma (MM).^7^ When treating myeloma or lymphoma involving the central nervous system (CNS), this drug can pass across the blood-brain barrier.^8,9^ Pomalidomide has been shown to enhance the TME and control T-cell activity. Pomalidomide has been demonstrated in preclinical investigations to upregulate bone marrow–derived dendritic cells (DCs) with major histocompatibility complex (MHC) class-I and CD86 molecules (CD86), hence enhancing antigen absorption and representation by DCs to naive CD8^+^ T-cells. Pomalidomide can also enhance MHC class II molecule expression on DCs, which raises CD4^+^ T-cell priming.^10^ Wang et al^11^ observed that in vitro pomalidomide treatment exhibited the ability to enhance the release of interferon (IFN)-γ by syndecan 1 (SDC1/CD138)-CAR-T cells. The aforementioned mechanisms might enhance the cytotoxic effects of CAR-T cells when directed at MM cell lines.^11^ In addition, various research has indicated that in vitro treatment of pomalidomide enhances the cytotoxic impacts of CAR-T cells targeting solid tumors. Moreover, it may also increase the release of cytokines by CAR-T cells.^12^ Given the promoting impact of pomalidomide on immune cells and the TME, it was speculated whether it could also promote the killing activity of CD19-CAR-T and BCMA cells.

It has been shown that 6 cases of RR hematological malignancies with extramedullary involvement were jointly provided with CAR-T cell therapy and pomalidomide for the first time. In this study, the possible in vitro regulatory effects of pomalidomide on BCMA- and CD19-CAR-T cells were examined, specifically in relation to their efficacy against hematological tumors. The findings indicate that pomalidomide exhibits the ability to enhance proliferation of T cell, sustain the anti-tumor effect of CAR-T cells, and stimulate the release of cytokines IFN-γ and tumor necrosis factor (TNF)-α.

## 2. MATERIALS AND METHODS

### 2.1. Patients

Six patients were retrospectively reviewed with EMD from 4 clinical trials in the Shanxi Bethune Hospital and Tongji Hospital treated between December 2020 and September 2023. These protocols (ChicTropc-16009113, ChicTR-OPN-16008526, ChiCTR-OPN-16009847, and NCT 05618041) were approved by institutional review boards and independent ethics committees of participating institutions (the ethical approval numbers are YXLL-2019-114 and YXLL-2022-001) before treatment. Three had RRMM, 2 had B-cell acute lymphoblastic leukemia (B-ALL), and 1 had B-cell non-Hodgkin lymphoma (B-NHL). All patients had EMD, including 2 cases with CNS invasion and 1 case with pericardial involvement. The study procedures underwent approval by institutional review boards and independent ethics committees of the participating institutions prior to the commencement of the treatment. The investigations were done in accordance with the guidelines set out by the International Conference on Harmonisation of Good Clinical Practice and the principles outlined in the Declaration of Helsinki. Informed consent was acquired from all participants to publish individual patient data.

### 2.2. CAR-T cell construction, generation, infusion, and kinetics

The BCMA-CAR-T cells were created using a lentiviral plasmid containing an anti-BCMA mouse-derived single-stranded variable region, which comprises a CD8 subunit alpha (CD8A) hinge, CD28 molecule (CD28) transmembrane region, intracellular domain, and CD247 molecule (CD247/CD3ζ) T cell activation domain.^13^ The third-generation CAR-T cells were used for targeting the CD19/CD22 molecule (CD22), which comprises a single-chain variable fragment (ScFv) derived from a murine monoclonal antibody against human CD19 or CD22, 2 costimulatory domains from CD28 and TNF receptor superfamily member 9 (TNFRSF9/4-1BB), and the activation domain from CD3ζ.^14^

All enrolled patients underwent peripheral blood mononuclear cell collection and CD3^+^ T-cell isolation in advance, followed by collection and freezing of CAR-T cells. All participants, with the exception of case 6, underwent lymphodepletion conditioning prior to CAR-T cell infusion, which involved the administration of fludarabine (30 mg/m^2^, days −4 to −2) and cyclophosphamide (300 mg/m^2^, days −4 to −2). Case 6 received the pretreatment BEAMF regimen (carmustine, etoposide, cytarabine, melphalan, and fludarabine) before a combination of CD19/22-CAR-T cell cocktail treatment and autologous stem cell transplantation (ASCT). The quantification of CAR-T cell growth in vivo was conducted using droplet digital polymerase chain reactions (PCR), as previously described.^15^

### 2.3. Procedures

Three patients with RRMM received radiotherapy as bridging therapy before CAR-T cell infusion, 1 with whole brain radiotherapy (2 Gy/time, 15 times in total, for a total of 30 Gy) and 2 with helical tomographic intensity-modulated radiotherapy of the extramedullary soft tissue (4 Gy/time, 5 times in total, for a total of 20 Gy). Treatment of case 6 was done, as mentioned previously. The administration of pomalidomide at a dosage of 1 to 2 mg occurred either on the same day as the infusion of CAR-T cells or following the CAR-T cell infusion, specifically when there was a drop in the copy number of CAR-T cells. Pomalidomide was discontinued with disease progression or unacceptable toxicity.

### 2.4. Efficacy assessments and follow-up

Blood samples were obtained from the peripheral and/or bone marrow after the injection of CAR-T cells in accordance with the specified follow-up date and the requirements of the clinical trial protocols. Multiparameter flow cytometry was employed in this investigation to identify and quantify minimal residual disease in cerebrospinal fluid and bone marrow. The efficacy of the treatment was evaluated based on the criteria established by the International Myeloma Working Group.^16^ Adverse events (AEs) were graded using the Common Terminology Criteria for AEs 5.0.^17^ The evaluation of EMDs involved various imaging techniques such as positron emission tomography-computed tomography (PET-CT), CT scans, magnetic resonance imaging (MRI), and/or examination of cerebrospinal fluid. The severity of cytokine release syndrome (CRS) and CAR-T cell–related encephalopathy syndrome was determined according to the criteria established by the CAR-T-Cell Therapy–Associated Toxicity Working Group.^18^ All participants in the study were monitored until their death, loss to follow-up, or withdrawal of consent. The follow-up period extended until September 30, 2023.

### 2.5. Pomalidomide combined with CAR-T cell therapy in vitro

#### 2.5.1. Cells and compounds

In this study, patient-derived T cells were investigated alongside CD19-CAR-T cells, BCMA-CAR-T cells, as well as other human cell lines, including 8226 and U266 MM cell lines, Raji lymphoma cell line, and K562 chronic myeloid leukemia cell line. The compound pomalidomide was procured from MedChemExpress (HY-10984; Monmouth Junction, New Jersey). It was then dissolved in dimethyl sulfoxide (DMSO) of cell culture grade, obtained from Solarbio, at a concentration of 10 mM and stored at −80°C.

#### 2.5.2. Cytotoxicity assay

T cells (CD3^+^ CD4^+^ or CD3^+^ CD8^+^) were initially separated from the patient’s blood sample and subsequently exposed to a concentration of 50 μg/mL pomalidomide starting from day 1. Throughout the experiment, the number of cells and their capacity to survive were evaluated on a daily basis.

The U266 MM cell line was introduced into 24-well plates with a concentration of 5 × 10^6^ cells/mL per well (1 mL per well), treated with DMSO (control) or pomalidomide at different concentrations (10, 25, or 50 μg/mL), and the apoptotic percentage was then detected by flow assay after 5 hours. Briefly, the cell culture supernatant was harvested and treated with Annexin V–labeled fluorescein to stain apoptotic cells, which were then detected by flow cytometry.

The combination of pomalidomide with BCMA-CAR-T cells was evaluated to induce apoptosis in 8226 and U266 cells. CAR-T cells were cocultured with 8226 or U266 cells at an efficacy-to-target ratio of 1:1, treated with different pomalidomide concentrations as described above, and apoptosis was detected by flow assay after 5 hours.

#### 2.5.3. Cytokine secretion

Tumor cell lines (8226, U266, and Raji) or patient-derived T cells were inoculated in 24-well plates, and 2 structural effector cells (CD19- or BCMA-CAR-T cells) were added at an effector target ratio of 1:1. TNF-α and IFN-γ secretion into the culture supernatant was detected after coculturing for 24 hours. A negative control group was set up: the killing of target cells by the pomalidomide solvent DMSO and of K562 cells by CAR-T cells.

Pomalidomide was introduced into the U266 cell line at concentrations of 10, 25, or 50 μM and into the 8226 cell line at a dose of 2.5 μM. The cells were then cocultured for 24 hours. The supernatant was collected, and the quantities of cytokines were assessed using Perkin Elmer AlphaLISA kits (IFN-γ: AL217C; TNF-α: AL208C; colony-stimulating factor 2 [CSF2/GM-CSF]: AL216C). The mean of 3 replicates was used to express the concentration of each cytokine.

### 2.6. Statistical analysis

The statistical studies were conducted using GraphPad Prism software, version 9.5. Mean killing efficiencies and cytokine concentrations were compared between 2 groups using a *t* test or between more than 2 groups using an analysis of variance. The analysis of the results pertaining to the death of target cells was conducted using the FlowJo program, version 10. Three parallel groups are created for each test. The experiments were conducted with a minimum of 3 repetitions. Statistical significance was determined at *P* < .05.

## 3. RESULTS

### 3.1. Patients and baseline features

Between December 2020 and September 2023, 6 patients were enrolled in this study (Table 1), including 3 cases of RRMM, 2 cases of B-ALL, and 1 case of B-NHL. Prior to the injection of CAR-T cells, all patients exhibited a state of disease progression, with each individual experiencing EMD. The median age of the individuals under study was 49 years, with a range spanning from 16 to 67 years. Between 2 and 9 therapy sessions, the median was 4. All 3 patients with RRMM showed resistance to pomalidomide in previous chemotherapy. Two patients (33.33%) had received hematopoietic stem cell transplantation (HSCT) before this treatment, including auto-HSCT and allo-HSCT. One of the 6 patients was treated with CAR-T cell cocktail treatment in conjunction with auto-HSCT. Table 1 outlines the patients’ baseline clinical characteristics of CAR-T cell treatment combined with pomalidomide.

**Table 1:** Clinical information of the 6 patients.

Number	Sex	Age	Diagnosis	Genetic alteration	Invasion	Number of prior therapies	Previous HSCT/CAR-T	Dosage of CAR-T cells	POM	Combine RT	Efficacy evaluation	CRS*	ICANS†
1	Female	62	MM	1q21amp	CNS	9	No	BCMA: 3.25 × 10^6^/kg	D0: 2 mg	Yes	CR	1	0
2	Female	62	MM	del 17p 1q21amp t(4:14)	Submandibular gland parotid gland soft tissue lymph node	3	ASCT	BCMA: 5 × 10^6^/kg	D37: 1 mg	Yes	PR	0	0
3	Female	67	MM	1q21amp t(14:20)	Lung, intestine, esophagus soft tissue	5	No	BCMA: 2 × 10^6^/kg	D0: 1 mg	Yes	PR	0	0
4	Female	26	Ph^+^ B-ALL	t(9;22)	CNS	3	CAR-T	CD19: 3.59 × 10^6^/kg CD22: 4.13 × 10^6^/kg	D0: 2 mg	No	CR	1	0
5	Female	16	B-ALL	-	Pericardium	5	CAR-T Allo-HSCT	CD19: 2 × 10^6^/kg CD22: 2 × 10^6^/kg	D29: 1 mg	No	CR	1	0
6	Male	36	DLBCL	TP53	Stomach lymph node	2	Combined ASCT	CD19: 2.25 × 10^6^/kg CD22: 3.51 × 10^6^/kg	D0: 1 mg	No	CR	0	0

Allo-HSCT = allogeneic hematopoietic stem cell transplantation, ASCT = autologous hematopoietic stem cell transplantation, B-ALL = B-cell acute lymphoblastic leukemia, BCMA = B-cell maturation antigen, CAR = chimeric antigen receptor, CNS = central nervous system, CR = complete response, CRS = cytokine release syndrome, DLBCL = diffuse large B-cell lymphoma, ICANS = immune effector cell-associated neurotoxicity syndrome, MM = multiple myeloma, Ph = Philadelphia chromosome, POM = pomalidomide, PR = partial response, RT = radiotherapy.

* CRS: refer to CARTOX-10 criteria.^18^

† ICANS: refer to CARTOX-10 criteria.^18^

### 3.2. Treatment and outcome

**Figure 1.**
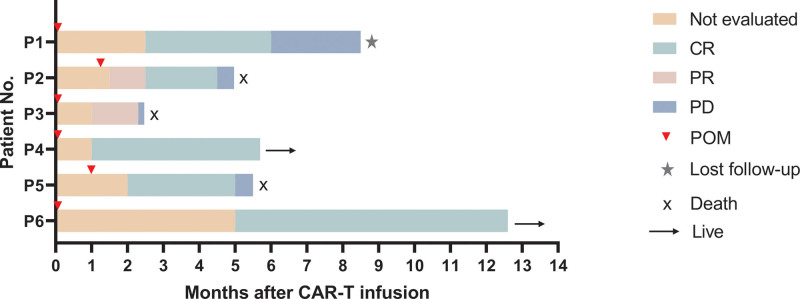
Clinical response to CAR-T cell therapy combined with pomalidomide. CAR = chimeric antigen receptor, CR = complete response, PD = progressive disease, POM = pomalidomide, PR = partial response.

All 6 patients had an overall response, as seen in Figure 1. Figure S1 http://links.lww.com/BS/A85, displays the therapy flowchart for the 6 patients.

#### 3.2.1. Relapsed and refractory multiple myeloma

**Figure 2.**
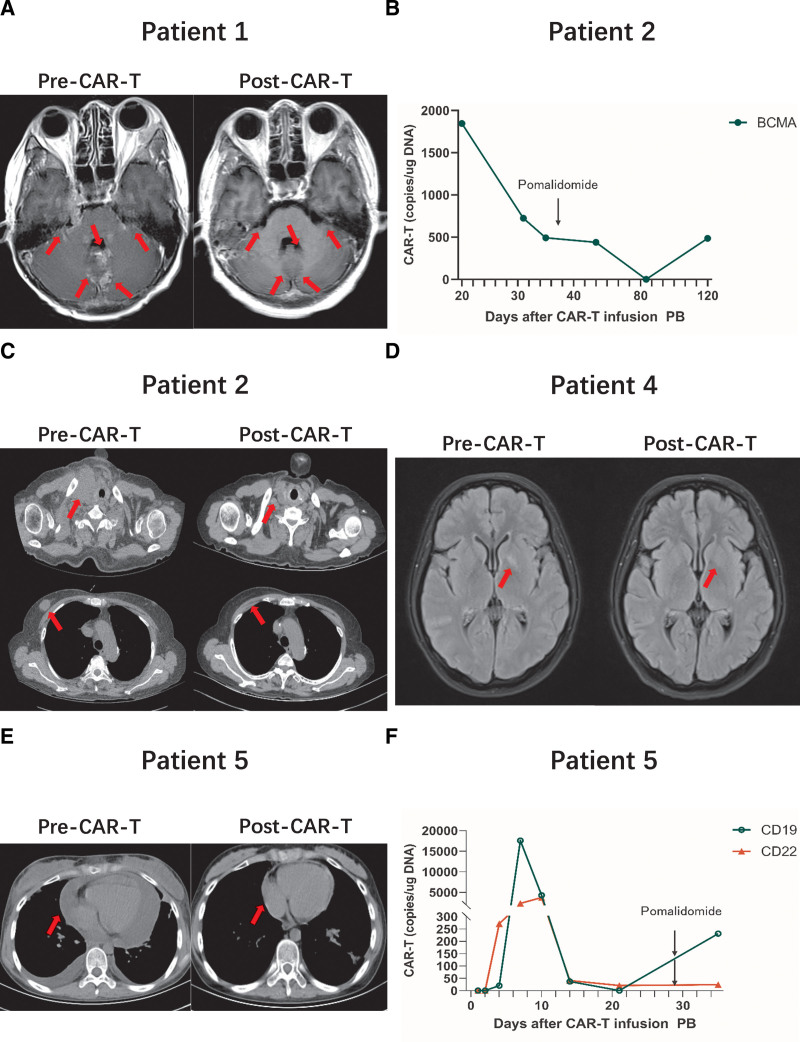
Summary of representative charts of patients during treatment. (A) Representative head MRI images of patient 1 before and after BCMA-CAR-T cell infusion. As shown in the MRI, the patient has extensive extramedullary lesions in the bilateral brain, cerebellum, midbrain, and pons of multiple myeloma. The lesions basically disappeared after 30 d of CAR-T cell infusion. (B) The trend of change in CAR-T cell copy number after infusion of BCMA-CAR-T cells in patient 2. On day 37 after infusion, the rate of decline in copy number slowed when pomalidomide was added. (C) Representative CT images of patient 2 before and after BCMA-CAR-T cell infusion. Those shown that patient 2 had lesions such as the left paramediastinum and left chest wall, which essentially disappeared after 45 d of CAR-T cell infusion. (D) Representative MRI images of patient 4 before and after CD19/CD22 CAR-T cells infusion. As was shown in MRI, she had lesions in the left basal ganglia, which completely disappeared after 30 d of infusion. (E) Representative CT images of patient 5 before and after CD19/CD22 CAR-T cells infusion. CT showed massive pericardial effusion from extramedullary infiltration of leukemia, which was completely absorbed 30 d after CAR-T cell infusion. (F) The trend of change in CD19/CD22-CAR-T cell copy number in patient 5 after CAR-T cell infusion, and the copy number bottomed out after the addition of pomalidomide on day 29 after CAR-T cell infusion. BCMA = B-cell maturation antigen, CAR = chimeric antigen receptor, CT = computed tomography, MRI = magnetic resonance imaging, PB = peripheral blood.

The best overall response rate (ORR) was 100% for the 3 patients with RRMM. In case 1, the brain MRI showed that the extramedullary lesions (bilateral temporal lobes; bilateral cerebellar brain hemisphere surfaces and sulci, pontine and midbrain surfaces; brainstem surfaces; intracerebral sulci; left cerebellar tonsil; and right insula) reached complete remission 1 month following the CAR-T cell therapy (**Fig. 2A**). In case 2, the observed decline in CAR-T cell copy number slowed after adding pomalidomide (**Fig. 2B**), and they eventually reached partial remission 1.5 months following the CAR-T cell injection (**Fig. 2C**). In case 3, the extramedullary lesions in multiple soft tissues and parenchymal organs disappeared except for one in the lungs 1 month following the CAR-T cell treatment. The progression-free survival (PFS) was 136 days (70–179 days) with a median follow-up of 150 days (75–254 days).

#### 3.2.2. RR B-cell leukemia/lymphoma

The best ORR was 100% for the 3 patients with RR B-cell leukemia/lymphoma. All 3 cases achieved complete remission. In case 4, the bone marrow reached complete remission after 14 days of infusion with CAR-T cells, and no leukemia cells were found in the lumbar puncture. Their abnormal lesions on MRI (left basal ganglia) disappeared after 30 days of CAR-T cell infusion (**Fig. 2D**). In case 5, CT showed massive pericardial effusion from extramedullary infiltration of leukemia, which was completely absorbed 30 days after CAR-T cell infusion (**Fig. 2E**). Pomalidomide was added when the CAR-T cell copy number decreased to 0, after which it increased again (**Fig. 2F**). Case 6 was examined by PET-CT at 5 months after infusion of CAR-T cells, showing the gastric and perigastric lymph nodes had all disappeared. With a median follow-up of 370 days, the OS of case 5 was 169 days; cases 4 and 6 remain alive.

### 3.3. Safety and AEs

There were no treatment-related deaths. CRS was reported in 3 patients (50%), manifesting as an increase in body temperature above 38°C without hypoxemia, hypotension, and organ dysfunction; all CRS were grade 1 (Table 1). No patient developed immune effector cell-associated neurotoxicity syndrome (ICANS). The AEs are listed in Table 2.

**Table 2:** Summary of the most commonly reported TEAE.

TEAE	Grade 1 n (%)	Grade 2 n (%)	Grade 3 n (%)	Grade 4 n (%)
Hematologic adverse events				
Anemia	0 (0)	2 (33.33)	4 (66.67)	0 (0)
Neutropenia	1 (16.67)	0 (0)	2 (33.33)	3 (50)
Thrombocytopenia	0 (0)	0 (0)	2 (33.33)	4 (66.67)
Prolonged APTT	0 (0)	0 (0)	0 (0)	0 (0)
Non-hematologic adverse events				
Hypoalbuminemia	2 (33.33)	3 (50)	1 (16.67)	0 (0)
Transaminitis	1 (16.67)	0 (0)	3 (50)	0 (0)
Electrolyte disorder	2 (33.33)	2 (33.33)	0 (0)	0 (0)
Hypertriglyceridemia	0 (0)	0 (0)	0 (0)	0 (0)
Hypoxia	0 (0)	0 (0)	0 (0)	0 (0)
Hypotension	0 (0)	1 (16.67)	0 (0)	0 (0)
Headache	0 (0)	0 (0)	0 (0)	0 (0)
Oral mucositis	3 (50)	0 (0)	0 (0)	0 (0)
Diarrhea	1 (16.67)	0 (0)	0 (0)	0 (0)
Tiredness	2 (33.33)	1 (16.67)	0 (0)	0 (0)
Dizziness	1 (16.67)	0 (0)	0 (0)	0 (0)
Tremor	0 (0)	0 (0)	0 (0)	0 (0)
Rash	1 (16.67)	0 (0)	0 (0)	0 (0)
Nausea	2 (33.33)	0 (0)	1 (16.67)	0 (0)
Severe adverse events				
Infection	0 (0)	3 (50)	2 (33.33)	0 (0)
Sepsis	0 (0)	0 (0)	0 (0)	0 (0)
Acute heart failure	0 (0)	0 (0)	0 (0)	0 (0)
Acute kidney injury	0 (0)	0 (0)	0 (0)	0 (0)

APTT = activated partial thromboplastin time, TEAE = treatment emergent adverse events.

Hematologic AEs were the most common severe AEs. All patients experienced pancytopenia attributable to the chemotherapy they received in this treatment and the extensive therapy they had received before protocol enrollment. It was clearly more prolonged than expected from the chemotherapy they received, especially in the patients with severe pretreated RRMM. Nevertheless, it should be noted that 3 patients with RRMM had pancytopenia before CAR-T cell therapy, 5 patients (83.33%) had grade ≥3 neutropenia, and all patients (100%) had grade ≥3 thrombocytopenia. Hematological toxicities were observed after 2 months in the patients with RRMM but not in the patients with RR B-cell leukemia/lymphoma.

### 3.4. Effects of pomalidomide with and without CAR-T cells on non-transfected T cells in vitro

**Figure 3.**
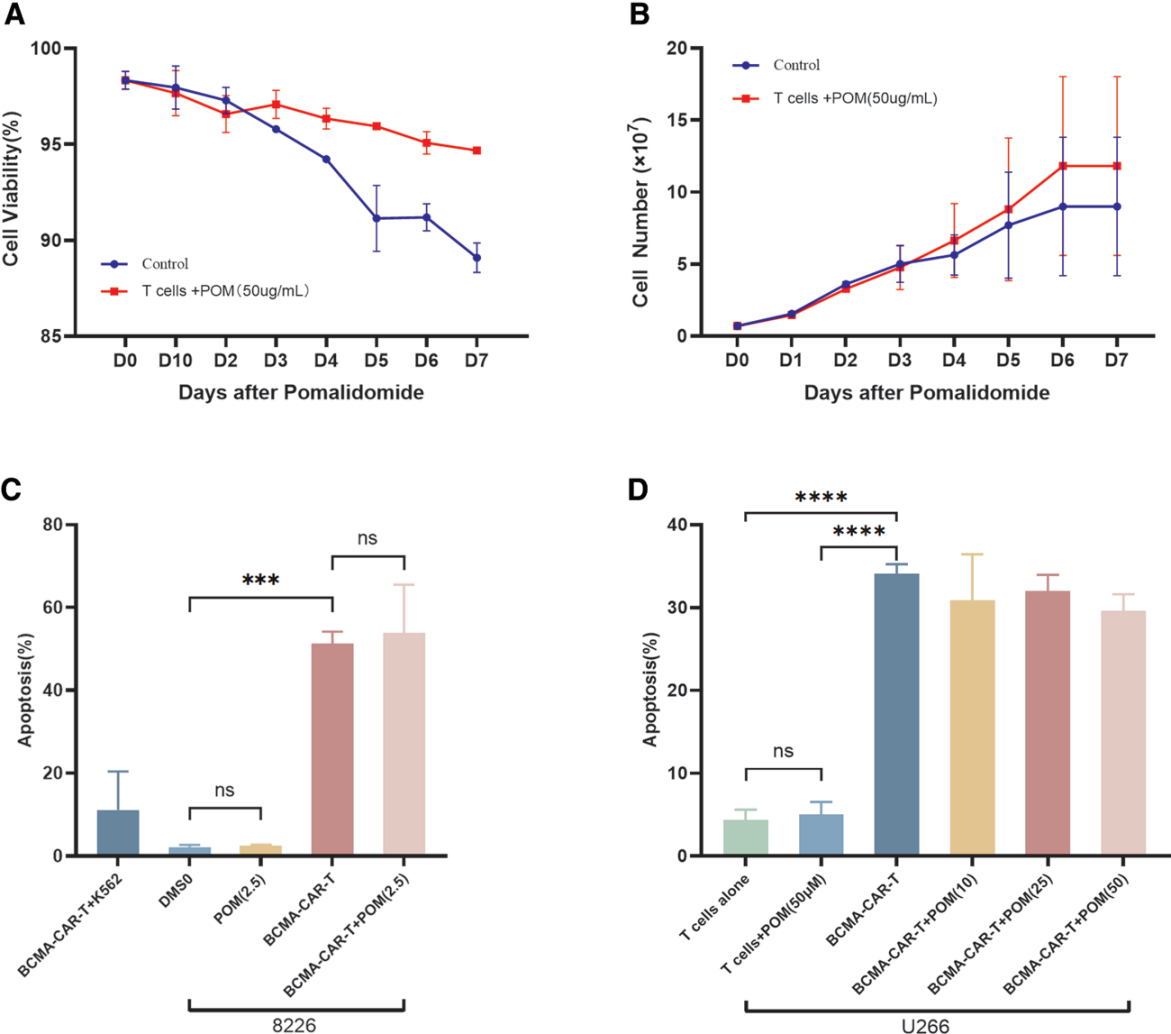
The expansion of T-cells and apoptosis of tumor cells were determined by flow cytometry. (A and B) Changes in the viability and number of T cells after combined with POM (50 µg/mL). (C) Apoptosis rate of 8226 cell lines treated by BCMA-CAR-T cell therapy combined with POM (2.5 µg/mL). (D) Apoptosis rate of U266 cell lines treated by BCMA-CAR-T cell therapy combined with POM of different concentrations. BCMA = B-cell maturation antigen, CAR = chimeric antigen receptor, DMSO = dimethyl sulfoxide, IFN = interferon, ns = no significance, POM = pomalidomide. ***P < .001; ****P < .0001.

Pomalidomide maintained T-cell viability and promoted cell expansion compared to the control treatment (**Fig. 3A, B**). MM U266 cell lines treated with different pomalidomide concentrations did not show significantly greater apoptosis than those treated with the control treatment at 5 hours (Fig. S2, http://links.lww.com/BS/A85), suggesting that pomalidomide has no killing effect on the U266 cells. However, BCMA-CAR-T cells showed a significant killing effect on MM 8226 cells (*P* < .05; **Fig. 3C** and Fig. S3, http://links.lww.com/BS/A85). On 8226/U266 cells, pomalidomide with CAR-T cells did not substantially outperform CAR-T cells alone (*P* > .05; **Fig. 3C, D** and Figs. S3 and S4, http://links.lww.com/BS/A85). Therefore, the results indicate that adding pomalidomide preserved the capacity of CAR-T cells to eliminate target cells.

Further investigation was conducted to examine the impact of varying doses of pomalidomide on the cytotoxicity of BCMA-CAR-T cells. The only DMSO control group had no discernible impact on the cytotoxicity of target cells. Additionally, varying doses of pomalidomide did not provide any statistically significant alterations in the cytotoxicity exerted by BCMA-CAR-T cells on U266 cells (Fig. S4, http://links.lww.com/BS/A85). There was no statistically significant variation observed in the impact of varied doses of pomalidomide on the cytotoxicity of CAR-T cells. In the context of this study, it was observed that the administration of pomalidomide did not result in a statistically significant increase in the cytotoxicity of normal T cells against U266 cells (*P* > .05). This finding suggests that pomalidomide does not exert an influence on the inherent killing capacity of CAR-T cells.

### 3.5. Pomalidomide induces CAR-T cell TNF-α and IFN-γ secretion

**Figure 4.**
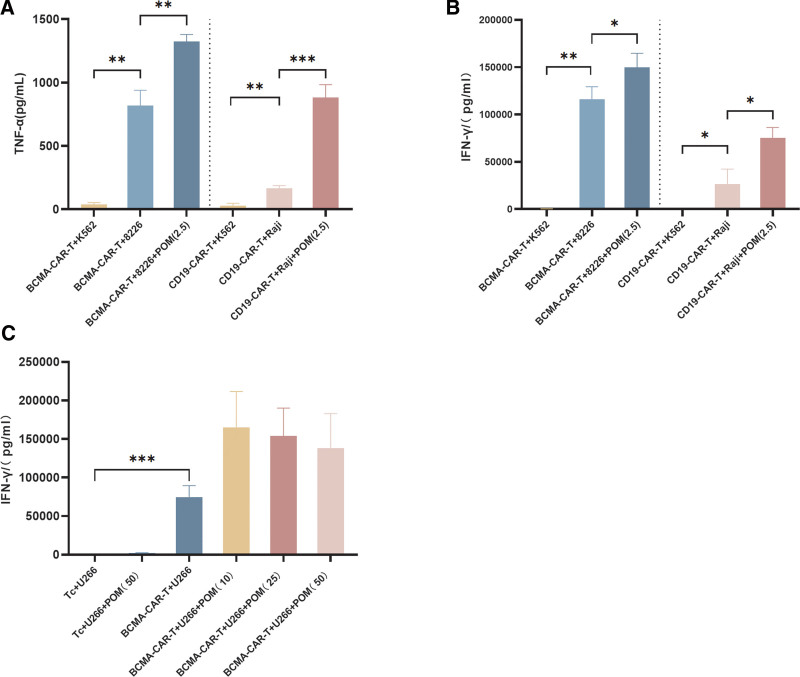
Cytokine secretion of IFN-γ and TNF-α was determined by flow cytometry. (A and B) Effect of BCMA-CAR-T cells combined with pomalidomide (2.5 µg/mL) on the 8226 cell line and CD19-CAR-T cells combined with pomalidomide (2.5 µg/mL) on the Raji cell line, cytokine secretion of IFN-γ and TNF-α was determined. (C) Effect of BCMA-CAR-T cell therapy and different concentrations of pomalidomide on the release of cytokine IFN-γ in U266 cell line. BCMA = B-cell maturation antigen, CAR = chimeric antigen receptor, CD = cluster of differentiation, IFN = interferon, POM = pomalidomide, Tc = T cells, TNF = tumor necrosis factor. *P < .05; **P < .01; ***P < .001.

BCMA- and CD19-CAR-T cells induced significantly greater TNF-α and IFN-γ secretion than the control K562 cells (*P* < .05; **Fig. 4A, B**). The cytokine production of CAR-T cells was seen to significantly increase (*P* < .05) when cocultured with tumor cells (U266, 8226, and Raji) in the presence of pomalidomide. To investigate this effect further, BCMA-CAR-T cells were subjected to cocultivation with U266 cells, along with various concentrations of pomalidomide (10, 25, and 50 μM). CAR-T cells grown with U266 cells with or without pomalidomide secreted almost no IFN-γ. CAR-T cell IFN-γ secretion was greater with pomalidomide (10, 25, or 50 μM) than without pomalidomide, with its secretion highest with 10 µg/mL pomalidomide (**Fig. 4C**).

## 4. DISCUSSION

This study administered CAR-T cell therapy with pomalidomide in 6 cases of RRMM or RR B-cell leukemia/lymphoma with EMD. Three of these patients had already undergone extensive pretreatment beyond the fifth line in the early stages. The ORR was 100% (4 had a complete response, and 2 had a partial response), and no grade ≥2 CRS or ICANS were observed, with a good safety profile. Importantly, the findings showed that pomalidomide did not affect CAR-T cell cytotoxicity. Furthermore, pomalidomide significantly enhanced CAR-T cells’ TNF-α and IFN-γ secretion. The study’s overall conclusions suggest that pomalidomide with CAR-T cell therapy might be a useful therapeutic combination for the treatment of hematological malignancies.

Pomalidomide has the ability to traverse the blood-brain barrier, as evidenced by recent in vitro investigations. This research has further demonstrated the synergistic outcomes resulting from the combination of pomalidomide with CAR-T cell treatment.^11^ However, no clinical reports have examined treating hematological malignancies with pomalidomide following CAR-T cell infusion. Zhang et al^19^ documented a clinical case involving a patient diagnosed with CNS-MM who underwent 2 cycles of pomalidomide-based bridging treatment prior to receiving sequential CAR-T treatment. The patient demonstrated a robust full response in relation to the sustained presence of CAR-T cells in patients for a minimum duration of 151 days. There is a potential for pomalidomide to augment the effectiveness of CAR-T cells. In the present study, a notable distinction was seen wherein the administration of pomalidomide occurred either on the day of or subsequent to the infusion of CAR-T cells, as opposed to before the initiation of CAR-T cell treatment.

In the conducted study, it was seen that all 6 patients exhibited positive responses to the administration of pomalidomide in conjunction with CAR-T cell treatment. Notably, 2 patients diagnosed with B-ALL had a subsequent infusion of CAR-T cells targeting the same antigen, resulting in the attainment of full remission. It was believed that combining CAR-T cell therapy with pomalidomide showed potential synergy. First, pomalidomide may promote CAR-T cell proliferation. When pomalidomide was added during the copy number decline period, a rebound copy number was observed in cases 2 and 5. Second, pomalidomide may have increased CAR-T cell efficacy. Prior research has demonstrated that the effectiveness of CAR-T cell treatment is dependent upon the administered dosage.^20^ For case 1, the suggested dose for BCMA-CAR-T cell infusion was 1 × 10^7^/kg.^21^ However, after 2 preparations, it was only 3 × 10^6^/kg, implying that T cells from heavily pretreated patients are of inferior quality and quantity. Nonetheless, they achieved complete remission after the combination treatment, indicating that even a limited CAR-T cell infusion had positive therapeutic effects in this patient, suggesting that CAR-T cell efficacy was possibly enhanced by pomalidomide. Moreover, the effectiveness of a subsequent infusion of CAR-T cells against the same target might be compromised. According to the findings of Gauthier et al,^22^ the administration of a second infusion of CD19-CAR-T cells in patients with RR B-cell malignancies led to a substantial decrease in the rate of full remission compared to the initial CAR-T cell infusion. The results of the multivariate analyses indicated that the introduction of cyclophosphamide-fludarabine lymphodepletion prior to the initial infusion of CAR-T cells, together with an increased dosage of CAR-T cells during the subsequent round of CAR-T cell treatment, were both significantly correlated with the attainment of sustained responses.^22^ The current research indicated that complete remission could be achieved with a second treatment of the same lymphodepletion regimen and CAR-T cell dose when pomalidomide was co-administered, implying that pomalidomide might enhance the effectiveness of a restricted amount of CAR-T cells.

This research aimed for examination of in vitro impact of pomalidomide on CAR-T cells. It indicated that BCMA-CAR-T cells had an apparent killing effect on MM cell lines and that adding pomalidomide did not affect BCMA-CAR-T cell cytotoxicity. The observed impact was shown to be statistically independent of the concentration of pomalidomide (*P* > .05), indicating that the presence of pomalidomide did not impede the cytotoxic activity of CAR-T cells. The fundamental principle underlying CAR-T cell therapy is the induction of tumor cell death through direct interactions between T cells and tumor cells. Nevertheless, the anti-tumor effectiveness of activated CAR-T cells may be enhanced through the secretion of cytokines, given the crucial function that these molecules execute in promoting the eradication of tumor cells via secondary mechanisms. In a study, Wang et al^12^ observed that the administration of pomalidomide resulted in the enhanced production and release of 4 different cytokines by CAR-T cells. The ability of CAR-T cells to combat solid tumors may be influenced by this modulation of cytokine secretion involving prominin 1 (PROM1/CD133) and erb-b2 receptor tyrosine kinase 2 (ERBB2/HER2) receptors.^12^ Additionally, in vitro experimentation demonstrated that pomalidomide significantly increased IFN-γ and TNF-α secretion by BCMA- and CD19-CAR-T cells, potentially explaining why CAR-T cells were more effective.

Three patients with RRMM included in this study received radiotherapy before CAR-T cell treatment. Multiple studies have documented that the integration of radiation and CAR-T cell treatment has yielded enhanced tumor infiltration and efficacy of CAR-T cells inside the TME.^23–26^ However, in this study, the primary goal of the initial radiation treatment was symptom relief. For example, case 1 had undergone heavy pretreatment and was resistant to current new drug treatments and chemotherapy, and they required emergency radiotherapy to relieve symptoms due to apparent intracranial hypertension. Emergency radiotherapy was also administered to cases 2 and 3 to treat their severe pain from large extramedullary masses and compression symptoms. The radiation therapy dose and schedule administered to these three individuals varied. The efficacy of CAR-T cell treatment may exhibit variability contingent upon the division of dosage and the temporal administration of radiation. Further investigation is required in order to determine whether the co-administration of radiation and CAR-T cell therapy produces a synergistic effect.

During combined pomalidomide therapy, there were no CRS or ICANS with grade ≥2, regardless of whether pomalidomide was administered concurrently with CAR-T cell infusion or the amount of CAR-T cells was decreased. Interestingly, grade 2 ICANS was observed during the initial administration of CAR-T cell in case 4. However, no associated ICANS was seen during the second treatment of the same CAR-T cells after CNS lymphoma and bone marrow relapse. Interestingly, 3 patients having administered CAR-T cell therapy subsequent to radiation did not experience grade ≥2 AEs while undergoing treatment.

There were various limitations inherent in the current study. The monitoring of CAR-T cell copy counts in 2 patients was not consistently performed. Nevertheless, the changes seen in the subpopulations of T-lymphocytes were indirect indications of the effectiveness of this combined therapeutic approach. For example, the CD8^+^:CD4^+^ T cells ratio in case 1 reached its maximum after CAR-T cell infusion and gradually decreased over time, while their B cells did not consistently recover to +D215 (Table S1, http://links.lww.com/BS/A85). Ding et al^27^ found that CAR-T cells had similar cell subset alterations to CAR^+^ cells. Secondly, the optimal dose and timing of pomalidomide administration remain unclear. While preclinical studies have demonstrated that the concurrent administration of immunomodulatory drugs and CAR-T cell infusion is more effective than their subsequent use, the optimal timing of pomalidomide infusion remains unexplored. Thirdly, while the present study showed that pomalidomide combined with CAR-T cell treatment was highly effective in individuals with RRMM or RR B-cell leukemia/lymphoma with EMD, it cannot ultimately prevent patients from disease regression, especially in RRMM. Therefore, for patients with high-risk factors, it may be more beneficial to administer pomalidomide in conjunction with the CAR-T cell treatment as a bridging therapy before infusion. Additional larger-scale randomized controlled studies are required to provide a more comprehensive assessment of the effectiveness and safety of the combination of pomalidomide and CAR-T cell therapy for the treatment of hematological carcinomas.

This finding indicates that pomalidomide failed to impair CAR-T cell functionality and enhanced the release of the cytokines. This research is the initial attempt to document the favorable effectiveness and manageable AEs resulting from the combination of CAR-T cell treatment with pomalidomide in individuals diagnosed with RRMM or RR B-cell leukemia/lymphoma accompanied by extramedullary involvement. Furthermore, it provided a new treatment strategy for hematologic malignancies with EMD.

## ACKNOWLEDGMENTS

This research was funded by the National Natural Science Foundation of China, grant number 82070217 (Jia Wei). This work was supported by 2023 COVID-19 Emergency Project of Shanxi Bethune Hospital (no. 2023xg02, Dr. Weiwei Tian) and Fundamental Research Program of Shanxi Province (no. 202303021211224, Weiwei Tian).

The authors acknowledge the team of staff and researchers at the Department of Hematology and Clinical Laboratory Center of Shanxi Bethune Hospital and Tongji Hospital for their assistance.

## ETHICAL APPROVAL

The four clinical trials in this study were approved by the institutional review board. This retrospective study was approved by the Medical Ethics Committee of the Department of Hematology, Shanxi Bethune Hospital, Shanxi Academy of Medical Sciences, Tongji Shanxi Hospital, Third Hospital of Shanxi Medical University, Taiyuan, Shanxi, and Department of Hematology, Tongji Hospital, Tongji Medical College, Huazhong University of Science and Technology, 430000 Wuhan, Hubei.

## AUTHOR CONTRIBUTIONS

All authors contributed to editorial changes in the manuscript. All authors read and approved the final manuscript. J.Z. and H.Y. participated in data collection, data analysis and drafted the initial manuscript. J.W. and W.T. designed the research and provided resources for the investigation. J.G. carried out in vitro experiments. J.W. and W.T. participated in reviews or editing of the manuscript. All authors contributed to the article and approved the submitted version.

## Supplementary Material


