# Supplementary material for: Pomalidomide improves the effectiveness of CAR-T treatment in the relapsed and refractory multiple myeloma or B-cell leukemia/lymphoma with extramedullary disease
Source: Blood Sci. 2024 Mar 1;6(2):e00184. doi: 10.1097/BS9.0000000000000184 (PMC10906647; doi:10.1097/BS9.0000000000000184)
Supplement: Supplementary file 1 [file bs9-6-e00184-s001.pdf]

Supplementary Material

This file includes:

Figures S1 to S4

Table S1

CATALOGUE

Supplementary Figures…………………………………………………...……...………1

Supplementary Figure 1. The flow chart of six patients treated with CAR-T therapy combined with pomalidomide……………………………………….……..……..……1

Supplementary Figure 2. Apoptosis rate of U266 cell line that cocultivated with different concentrations of pomalidomide………………………………..………………….………..3

Supplementary Figure 3. Apoptosis rate of 8226 cell line were cocultivated with pomalidomide in combination with BCMA-CAR-T cells…….......................................…………..….......4

Supplementary Figure 4. Apoptosis rate of U266 cell line were cocultivated with different concentrations of pomalidomide in combination with BCMA-CAR-T cells............................5

Supplementary Table…………………………………………………….…………..……6

Supplementary Table 1. Dynamic changes in lymphocyte subsets in patient 1 after CAR-T cells infusion in combination with pomalidomide. ……………………….………........…..6

1. Supplementary Figures

Supplementary Figures 1. The flow chart of six patients treated with CAR-T therapy combined with pomalidomide

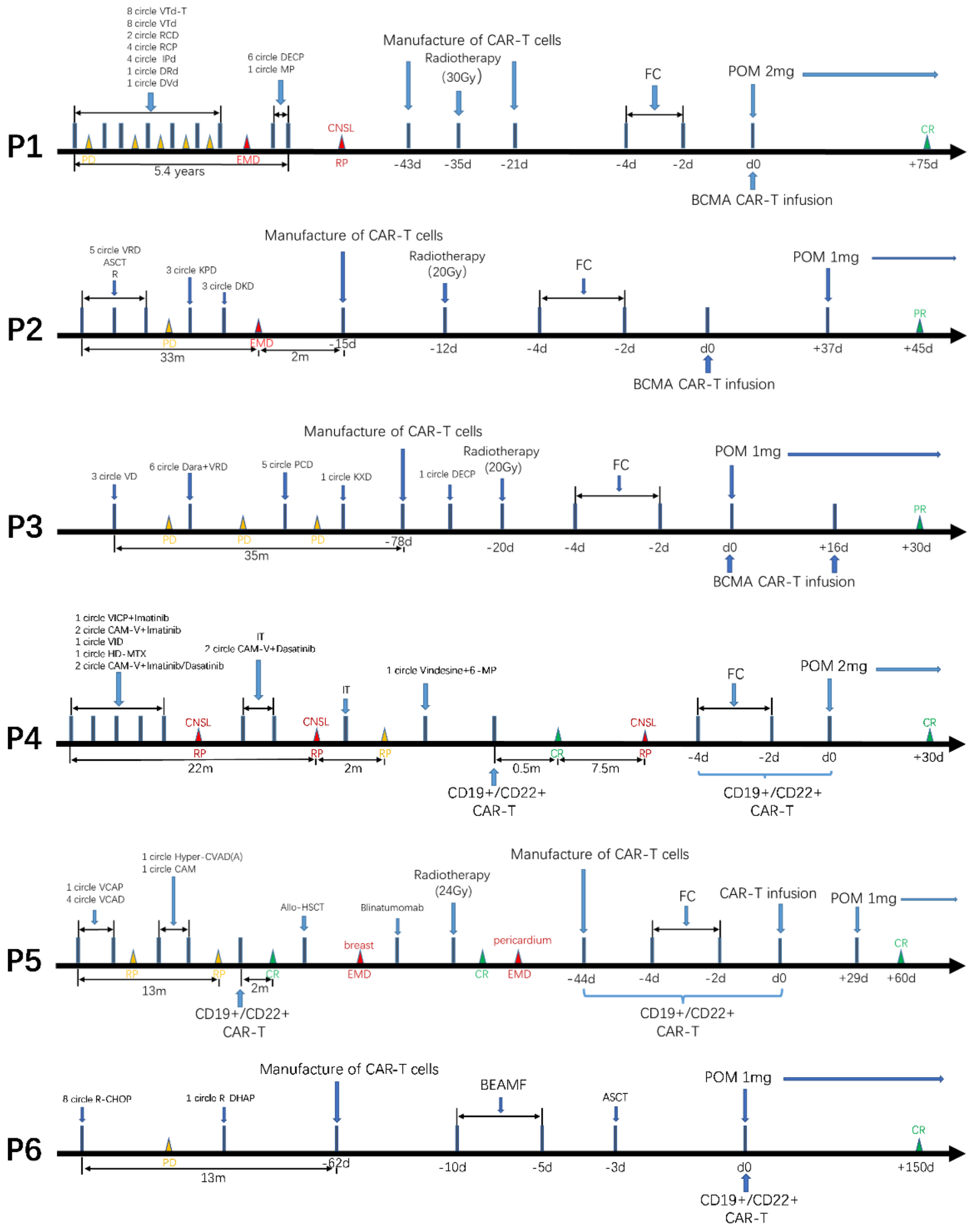

Abbreviations: VTD,bortezomib/thalidomide/dexamethasone; RCD,bortezomib/lenalidomide/dexamethasone; RCP,lenalidomide/cyclophosphamide/prednisone; IPD,ixazomib/pomalidomide/dexamethasone; DRd,daratumumab/lenalidomide/dexamethasone; DVd,daratumumab/bortezomib/dexamethasone; DECP,dexamethasone/etoposide/cyclophosphamide/cisplatin; MP,melphalan/prednisone; VRD,bortezomib/lenalidomide/dexamethasone;ASCT,autologous stem cell transplantation; KPD,carfilzomib/pomalidomide/dexamethasone; DKD,daratumumab/carfilzomib/dexamethasone; VD,bortezomib/dexamethasone; Dara,daratumumab; PCD,pomalidomide/cyclophosphamide/dexamethasone; KXD,carfilzomib/selinexor/dexamethasone; VICP,vinblastine/idarubicin/cyclophosphamide/prednisone; CAM-V,cyclophosphamide

/cytarabine/6-mercaptopurine/vinblastine; VID,vinblastine/idarubicin/dexamethasone;HD-MTX,high-dose methotrexate; IT,intrathecal injection; VCAP,vincristine/cyclophosphamide/daunorubicin/prednisolone; VCAD,vincristine/cyclophosphamide/daunorubicin/dexamethasone; Hyper-CVAD(A)=cyclophosphamide/doxorubicin/vincristine/dexamethasone; CAM,cyclophosphamide/cytarabine/6-mercaptopurine; HSCT,hematopoietic stem cell transplantation; R-CHOP,rituximab/cyclophosphamide/doxorubicin/vincristine/prednisone; DHAP,rituximab/dexamethasone/cytarabine/cisplatin; BCMA,B-cell maturation antigen; CAR,chimeric antigen receptor; EMD,extramedullary disease; CNSL,central nervous system leukemia;FC, fludarabine/cyclophosphamide; POM,Pomalidomide; RP,relapse; CR,complete response; PR,partial response; PD,progressive disease.

Supplementary Figure 2. Apoptosis rate of U266 cell line that cocultivated with different

concentrations of pomalidomide

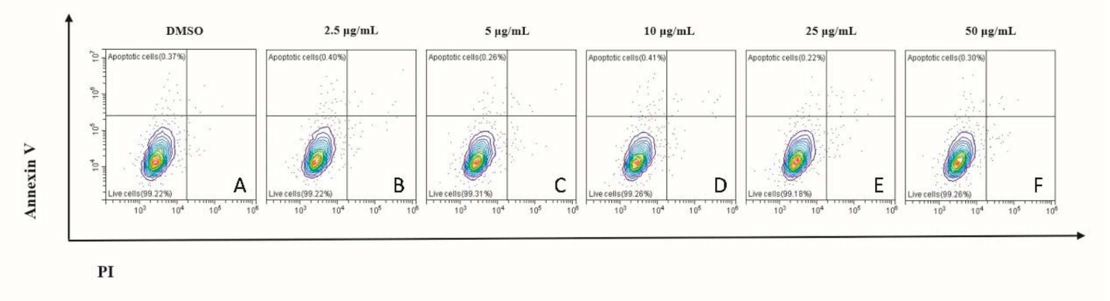

A: control; B: pomalidomide 2.5ug/mL; C: pomalidomide 5ug/mL; D: pomalidomide 10ug/mL; E: pomalidomide 25ug/mL; F: pomalidomide 50ug/mL.

Supplementary Figure 3. Apoptosis rate of 8226 cell line were cocultivated with

pomalidomide in combination with BCMA-CAR-T cells

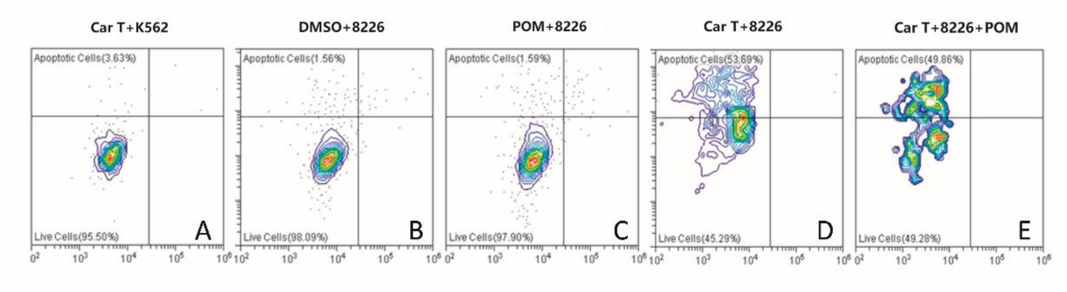

A: BCMA-CAR-T cell + K562 cell line; B: control; C: pomalidomide 2.5ug/mL + 8226 cell line; D: BCMA-CAR-T cell + 8226 cell line; E: BCMA-CAR-T cell + pomalidomide 2.5ug/mL + 8226 cell line.

Supplementary Figure 4. Apoptosis rate of U266 cell line were cocultivated with different

concentrations of pomalidomide in combination with BCMA-CAR-T cells

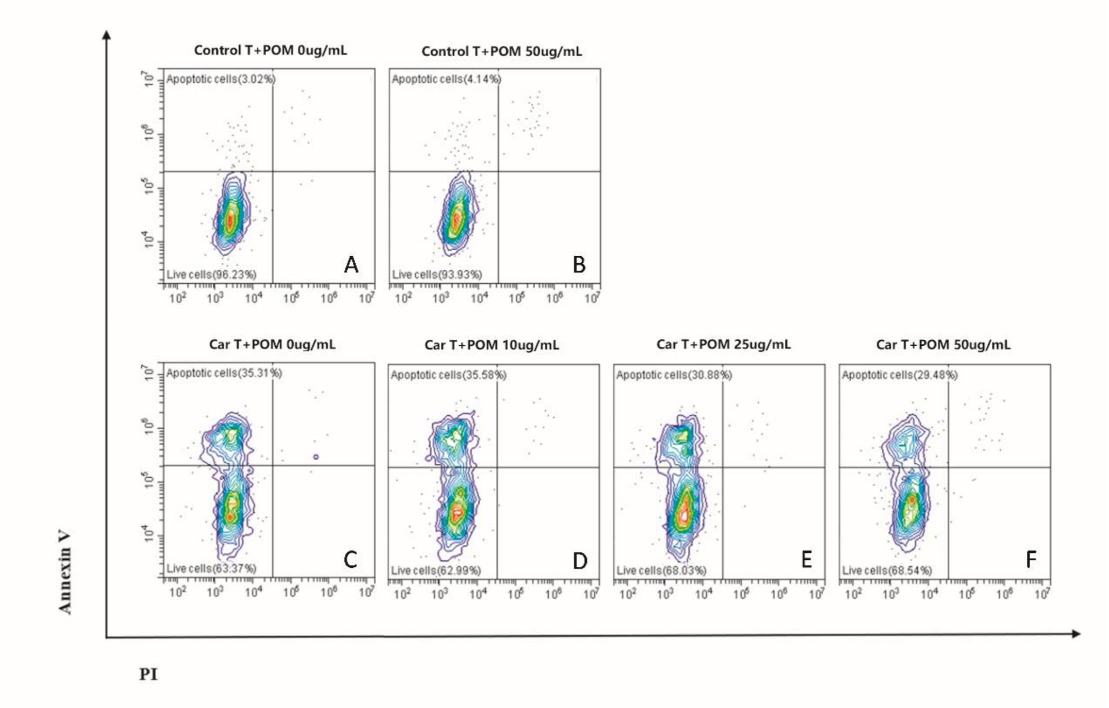

A: T cells + U266 cell line; B: T cells + U266 cell line + pomalidomide 50ug/mL; C: BCMA-CAR-T cells + U266 cell line; D: BCMA-CAR-T cells + U266 cell line + pomalidomide 10ug/mL; E: BCMA-CAR-T cells + U266 cell line + pomalidomide 25ug/mL; F: BCMA-CAR-T cells + U266 cell line + pomalidomide 50ug/mL. POM, pomalidomide.

2.Supplementary Table

Supplementary table 1. Dynamic changes in lymphocyte subsets in patient 1 after CAR-T cells infusion in combination with pomalidomide

Day	-D11	D0	+D4	+D10	+D20	+D45	+D77	+D141	+D215
CD4+ Th (/ul)	599	14	8	127	143	159	162	123	113
CD8+ Ts (/ul)	866	13	21	302	282	247	185	62	33
CD4/CD8 (%)	0.69	1.08	0.38	0.42	0.51	0.64	0.88	1.98	3.42
B-cell (/ul)	94	0.2	0	58	23	2	0	0	6
